# Unusual F-18 FDG Uptake at the Ears of a Patient with Discoid Lupus Erythematosus Diskoid Lupus Eritamatozus

**DOI:** 10.4274/Mirt.021793

**Published:** 2012-04-01

**Authors:** Tamer Özülker, Filiz Özülker, Filiz Cebeci, Adnan Somay, Mehmet Tarık Tatoğlu, Tevfik Özpaçacı

**Affiliations:** 1 Okmeydanı Educational and Research Hospital, Department of Nuclear Medicine, İstanbul, Turkey; 2 Bezmialem Vakıf University Faculty of Medicine, Department of Dermatology, İstanbul, Turkey; 3 Bezmialem Vakıf University Faculty of Medicine, Department of Pathology, İstanbul, Turkey

**Keywords:** Positron-emission tomography, lupus erythematosus, discoid, fluorodeoxyglucose F-18

## Abstract

A 55-year-old patient, who had undergone excisional biopsy of upper lip two years ago and diagnosed to have squamous cell carcinoma, was referred to us for evaluation with Fluorine-18 fluorodeoxyglucose (F-18 FDG) positron emission tomography (PET)/CT (F-18 FDG-PET/CT) scan. F-18 FDG-PET/CT scan was performed and the maximum intensity projection images (MIP) showed unusual FDG uptake at both ears. Histopathological examination of the biopsy specimen obtained from the ears revealed discoid lupus erythematosus (DLE).

**Conflict of interest:**None declared.

## INTRODUCTION

Discoid lupus erythematosus (DLE) is a chronic and inflammatory dermatosis which may progress to systemic lupus erythematosus (SLE). F-18 FDG-PET/CT has been shown to demonstrate inflammation via the increased glucose uptake ability of granulocytes and tissue macrophages (1). Here we present a case of DLE in which increased F-18 FDG uptake at the ears of a patient was detected probably because of this FDG avidity of inflammatory cells.

## CASE REPORT

**Figure 1:**
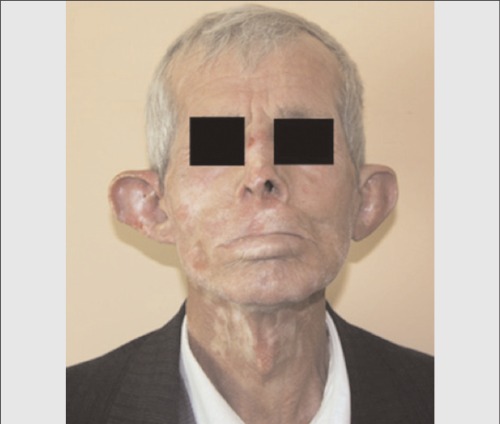
Patient with discoid lupus erythematosus

**Figure 2:**
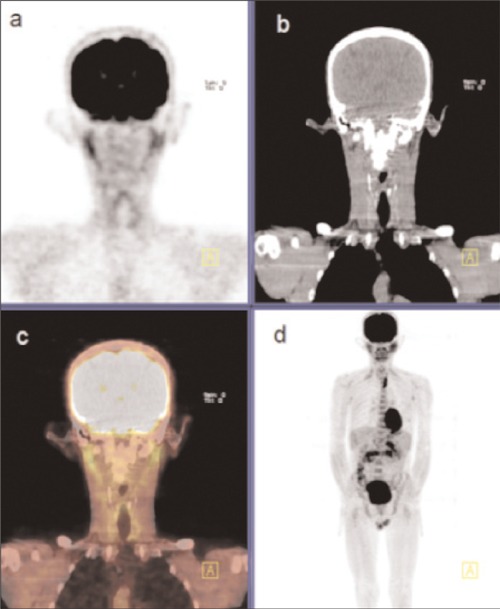
PET (a), CT (b), fusion (c) and MIP (d) images of the patientshowing unusual uptake at ears and nonspecific uptake at mediastinum

**Figure 3:**
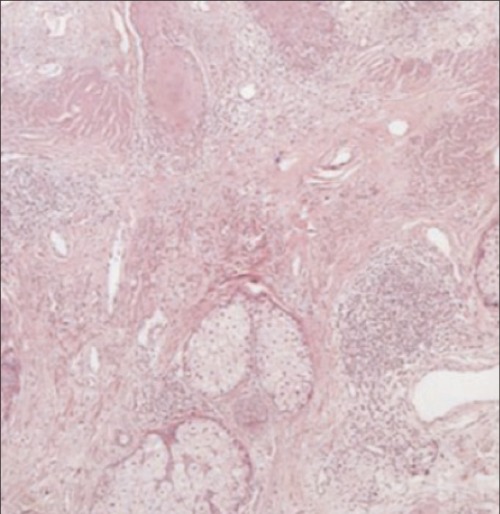
Histopathological examination of the biopsy specimenobtained from the ears showing hyperkeratotic epidermis andperivascular and perifollicular moderate inflammatory cell infiltrationat upper and medium dermis H&E x 100

A 55-year-old patient, who had undergone excisional biopsy of upper lip two years ago and diagnosed to have squamous cell carcinoma, was referred to us for evaluation with F-18 FDG-PET/CT scan. On physical examination, there were erythematous scaly lesions on the face, especially on the ears, telangiectasias on the cheek and rash on the bridge of the nose. Additionally, there were plaques, extending from the cheek to the neck, with scaly centers lighter in color and with dark edge (Figure 1). Anamnesis of the patient revealed that these lesions had been present for twenty-five years and the patient had used topical steroids irregularly. F-18 FDG-PET/CT scan was performed and unusual FDG uptake was detected at both ears. There was also nonspecific uptake at mediastinal lymph nodes (Figure 2). Histopathological examination of the biopsy specimen obtained from the ears revealed hyperkeratotic epidermis and perivascular and perifollicular moderate inflammatory cell infiltration at upper and medium dermis (Figure 3). Further laboratory examinations showed no evidence, suggesting SLE and the case was diagnosed as DLE with these clinical and histopathological findings.

## LITERATURE REVIEW AND DISCUSSION

The utility of F-18 FDG-PET/CT in patients with SLE has been limited to the central nervous system (2,3,4,5). In one study, PET scans showed hypometabolism in at least one brain region in all patients with severe or mild CNS symptoms (100%) and the authors decided that PET imaging represents a sensitive tool to detect manifest or subclinical CNS involvement in SLE and PET findings correlate well with the clinical course of disease (6). F-18 FDG-PET can identify fluctuations in regional cerebral metabolism in neuropsychiatric SLE (NP-SLE) patients, even when no structural lesions are evident on magnetic resonance imaging (MRI) (3). Computed tomography (CT) or MRI have been found to be useful in the detection of focal neurological deficits rather than diffuse presentations in patients with SLE (7,8,9). It was shown that FDG-PET could help to verify brain-onset of SLE earlier and it might also be a powerful tool for controlling SLE treatment (10). FDG-PET is shown to be more sensitive than MRI and CT in the diagnosis of NP-SLE, with its ability of demonstrating even reversible deficits and showing a better correlation with other neurological findings (11). DLE is an autoimmune inflammatory disorder of the skin that often leads to scarring and alopecia. While about 15% to 20% of patients with SLE manifest DLE lesions, only about 5% to 10% of patients with DLE go on to develop SLE (12). Classic DLE is the most common form of chronic cutaneous lupus erythematosus (CCLE) (13). Inflammatory cells have been reported to have an increased uptake of F-18 FDG (14) and this is likely to be the mechanism responsible for FDG uptake at the ears of our patient. Today, we do not have enough knowledge about the significance of this incidental FDG uptake at the ears. It has been reported that both patients with active lupus and those with inactive lupus had increased FDG uptake in lymph nodes when compared with healthy volunteers so F-18 FDG-PET/CT can not distinguish active and clinically quiescent disease (15). To our knowledge, this is the first case in the literature showing F-18 FDG uptake at lesions of DLE and further studies might be helpful to detect any possible role of F-18 FDG-PET/CT in the evaluation of the activity and severity of DLE.
